# Effects of a Social and Emotional Competence Enhancement Program for Adolescents Who Bully: A Quasi-Experimental Design

**DOI:** 10.3390/ijerph19127339

**Published:** 2022-06-15

**Authors:** Yul-mai Song, Sunah Kim

**Affiliations:** 1 Honam University, Gwangsan-gu, Gwangju 62399, Korea; 2 Yonsei University, Seodaemun-gu, Seoul 120749, Korea; psy0962@yuhs.ac

**Keywords:** bullying, violence, social and emotional competence, intervention, mental health

## Abstract

Background: The purpose of this study was to develop a social and emotional competence enhancement (SECE) program as an intervention for adolescents who bully, and to investigate its effects on school bullying behavior and mental health. Methods: A pre-posttest, 1-month follow-up nonequivalent control group quasi-experimental design was used. In total, 71 school bullies were included in the analysis. Results: The effects of this program were significant with regard to group-by-time interaction effects on social competence, emotional regulation, empathy, and school bullying behavior at the 1-month follow-up. Conclusions: The results indicate that the SECE program was effective at reducing school bullying behavior in adolescents who bully. School and community-based mental health professionals can provide feasible interventions that can be used in the short term to reduce school bullying behavior in adolescents who bully.

## 1. Introduction

School bullying is recognized as a serious social problem as well as a major problem during childhood [1]. Bullying is a collection of behaviors that can be characterized by the following: aggression or intent to harm, which is performed repeatedly over time and occurs in interpersonal relationships in which a power imbalance exists [2]. School bullying exerts serious physical, mental, and social effects in both the children and adolescents suffering from bullying and the children and adolescents who bully, who are more likely to experience health, property, and social relationship issues and engage in illegal behavior in adulthood [3,4,5]. A previous study predicted that adolescents who bully who do not receive appropriate help and support are associated with habitual criminal behavior in adulthood [6]. School bullying exerts a significant effect on mental health and psychosocial adjustment because it is consistently relevant regardless of location and is, therefore, a key component of mental illness [7].

Prevention is heavily emphasized in efforts to resolve school bullying, and in general, it has been centered on universal prevention for entire schools [8,9]. In a meta-analysis study conducted to confirm the effect of a school program based on social–emotional learning, it was verified that it is effective with respect to social–emotional skills, attitudes, positive social behavior, academic performance, and conduct problems [10,11,12]. In particular, it was found to be effective in reducing aggressive and destructive behavior and bullying in relation to conduct problems [13,14]. To obtain the positive benefits of social–emotional learning, a universal preventive strategy was provided for all classroom students, but in practical terms, it may be lacking in effectiveness [15].

Therapeutic approaches for children and adolescents who have already experienced school bullying are relatively inadequate compared to preventive strategies, and there are few therapeutic interventions available for adolescents who bully [16]. Individual factors that have been reported as being related to school bullying include gender (boys), age, weight (distant from average), physical health problems, self-esteem, anxiety, aggression, impulsivity, empathy, and social skills. Family factors have been found to be highly related to socioeconomic level, witnessing family violence, and experiences of abuse. As for school factors, academic performance, school maladjustment, delinquency experience, relationships with teachers, and relationships with friends were highly related [17]. Previous studies have shown that the behavior of bullying is not caused by one factor but, rather, occurs through the interaction of individual, family, school, and environmental factors [18]. In addition, adolescents who bully have been reported to be associated with mental disorders such as depression, conduct disorder, attention-deficit hyperactivity disorder (ADHD), learning disorders, and autism spectrum disorder, and they are typically also vulnerable subjects with problems relating to low self-esteem, high levels of aggression, impulsivity, low empathy, and lack of social skills [17,19,20,21,22]. Based on a literature review of 15 intervention studies conducted with children and adolescents who bully, key components of effective interventions were derived from self-awareness, emotional intelligence, interpersonal skills, and responsibility [23]. These key components are consistent with the social and emotional competence of social and emotional learning.

Social and emotional learning was established to support the social, emotional, and academic development of children and adolescents [24]. Social and emotional competence is the ability to understand and control one’s own thoughts, emotions, and behaviors and adapt to those of others to establish positive relationships and to make appropriate decisions to solve social life problems. It consists of five domains: self-awareness, self-management, social awareness, interpersonal skills, and responsible decision making [24]. Social and emotional learning programs are increasingly being implemented in schools to address a wide range of problematic behaviors and to promote prosocial behavior, academic success [10,11]. Social and emotional competence is known to exert a positive impact on young people’s aggressive behavior and promote mental health [25]. In a previous study, as a result of providing an emotional literacy program by first applying social and emotional learning to children who bully, school bullying behavior was reduced [26]. However, in that study, lower-grade elementary school children who bullied in school were selected via peer nomination, so they could be regarded as subjects of school bullying at a somewhat low severity level. The content of the program was also limited to emotional literacy, focusing on recognizing, understanding, processing, and appropriately expressing emotions.

The current study was conducted to develop a social and emotional competence enhancement (SECE) program—a program in which all components of social and emotional competence are integrated—and to confirm its effect on severe cases of adolescents who bully. The aforementioned literature review demonstrated that an SECE program could help adolescents who bully increase their social and emotional competence, thereby reducing school bullying behavior and promoting mental health. The hypotheses of this study are as follows:

**Hypothesis** **1.** *The experimental group subjected to the SECE program will show a greater increase in social and emotional competence relative to the control group.*

**Hypothesis** **2.** *The experimental group subjected to the SECE program will show a greater reduction in school bullying behavior relative to the control group.*

**Hypothesis** **3.** *The experimental group subjected to the SECE program will show a greater increase in mental health relative to the control group.*

## 2. Materials and Methods

### 2.1. Research Design

We used a quasi-experimental, non-randomized design to verify the effects of our SECE program on school bullying behavior and on the mental health of adolescents who bully.

### 2.2. Participants

The participants were adolescents who bully who were provided with compulsory education for school bullying at one of the Ministry of Justice’s educational institutions in City D, South Korea. The institution conducts compulsory education as requested by schools, prosecutors, and courts for adolescents who bully in clear and serious bullying situations. This means that bullying is observed by witnesses (teachers, school staff, other students) at the school or reported by the victims, and the bullying is confirmed through investigation by the school committee or the police. The compulsory education conducted at the institution is commuter-style, that is, without lodging, from 9 a.m. to 5 p.m. for a total of 5 days. The main contents of compulsory education consisted of legal procedures for punishment and education for prevention of violence. The inclusion criteria were (1) age of 12–16 years and attendance at middle school, (2) no problems with communication due to physical and mental difficulties, and (3) cases in which school bullying was observed by institutions (schools, prosecutors, courts) within the last 12 months. The exclusion criteria were as follows: (1) has ADHD or learning disabilities (this requires a different approach) and (2) is currently receiving psychiatric treatment including medication (to ensure exclusion of psychiatric treatment effects). Based on a covariance analysis, G-power 3.1 (Kiel, Germany) [27] was used to determine the sample size, with a large effect size of 0.4, a power of 0.80, and a significance level of 0.05. Considering the dropout rates in previous studies involving adolescents [28], we predicted a rate of 10% and recruited 72 participants.

### 2.3. Procedures and Data Collection

This study was conducted from March to December 2018. At the participating institutions, we explained the study to the headmaster and teachers. The chosen institution was selected in consideration of the convenience and accessibility of data collection among the potential institutions that could recruit participants that meet the purpose of this study. The investigator visited the institution and recruited the participants directly. The recruitment method involved attaching a poster in the form of a picture in the resting space of the institution and providing a brief introduction and guide during a break in the compulsory education. Adolescents who bully who agreed to participate in the study contacted their guardians, such as a parent, and obtained consent to participate in person or by wire. The experimental group and the control group assignments were based on the week the participants began the compulsory education, with the experimental group assigned to the even-numbered weeks and the control group assigned to the odd-numbered weeks. The institution where the study was conducted is a place where compulsory education is provided to an average of 15 adolescents who bully for 5 days, and in order to determine the feasibility of the experiment and prevent the spread of the experimental effect, random assignment was not used.

The SECE program (10 sessions) developed in this study was intensively provided for 5 consecutive days. Data were collected three times, a pretest was conducted on the first day of compulsory education, which was a 5-day course, and then the first session of the program was conducted. The final session of the program and the first posttest were conducted on the last day of compulsory education. One month after the end of the program, the second posttest was conducted. The pretest and first posttest were collected face-to-face, but the second posttest (after 1 month) was collected during daily life, so a web survey was conducted to enable data collection anywhere. A survey was created using Google Docs, and the survey URL was sent to a contact (mobile phone number or e-mail address) that was obtained beforehand. To increase the response rate of the web survey, a push message was sent regularly. In addition, in consideration of the participant’s privacy, no personal information other than the subject ID was recorded in the web survey.

**Figure 1:**
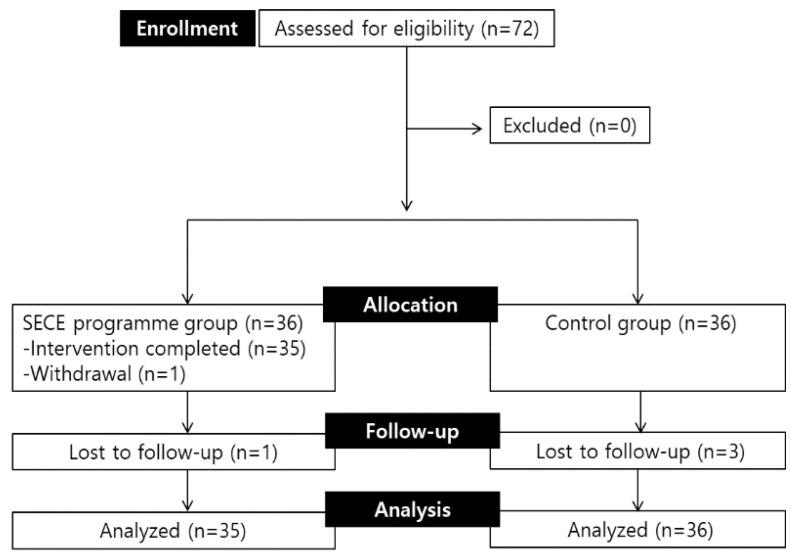
Flow diagram for the study. SECE—social and emotional competence enhancement.

Data collection for the control group was performed in accordance with that for the experimental group. On the first day of compulsory education, we conducted a pretest and school bullying prevention education. The survey method and content were provided in the same manner as that for the experimental group. All programs provided to the experimental group and the control group were conducted by the investigators, and the pretest and first posttest were conducted by two research assistants trained for this study to ensure the independence of intervention and investigation. Figure 1 depicts a flow diagram illustrating the recruitment and participation process. Seventy-two participants were recruited, 36 each in the experimental group and the control group. However, one participant in the experimental group was excluded due to their intention to withdraw during the program, and one did not respond to the first posttest. All control participants completed until the first posttest; however, three did not respond to the second posttest. Therefore, a total of 67 participants, including 34 in the experimental group and 33 in the control group, provided data after completing all three assessments. However, the linear mixed model (LMM), the analysis method used in this study, can be included in the analysis even if there are missing values, so a total of 71 subjects’ data were analyzed, excluding the data of one participant who did not participate in the program.

### 2.4. Ethical Considerations

The study protocol was approved by the institutional review board of the institute with which the authors are affiliated and conformed to the ethical standards stipulated in the Declaration of Helsinki (NCMH-116271-2018-37). To protect the participants’ rights, the study objective and procedures were explained beforehand. Because of the vulnerable nature of the adolescent population, the purpose of the study was also explained to their family members or legal representatives, from whom written consent was obtained. Data were statistically processed through serial identification numbers and stored in a locked laboratory that could only be accessed by one investigator.

### 2.5. Intervention

The social and emotional competence enhancement (SECE) program was developed by the investigators in accordance with the Analysis, Design, Development, Implementation, and Evaluation model procedure [29]. The program was based on the social and emotional competence in the “Collaborative for Academic, Social, and Emotional Learning’’ [24] following a literature review, expert supervision, a pilot study, and appropriate modification and supplementation. In the analysis phase, the characteristics and interventions of adolescents who bully were reviewed and analyzed, and in the design phase, the contents, form, and delivery method of the program were set based on the results of a literature review. In the development phase, experts verified the validity of the composition and contents of the program, and this was reflected on and corrected as needed. In the implementation phase, a pilot study was conducted on three adolescents who bully in order to confirm the feasibility of the program prior to application, and in the evaluation phase, the program was supplemented and confirmed based on the results of the pilot study.

Group-type interventions are generally known to be a useful approach for adolescents, but they can exert a negative effect on adolescents with conduct problems [30]. Therefore, the program was developed by combining group and individual formats to ensure that it could be predicted and properly controlled in advance. The program included a total of 10 sessions lasting 45 min, which were provided twice per day intensively for 5 consecutive days. The first and final sessions consisted of face-to-face counselling as an individual intervention, while Sessions 2–9 involved group intervention with a cognitive behavior strategy. In the individual interventions, nursing counselling was conducted based on a therapeutic relationship focused on experiences of school bullying, motives of the bullying, the dynamics of the participant’s family, relationships with friends, unresolved difficulties, decision-making patterns, and reflection. Table 1 shows the program’s configuration, sessions, and content. Each session began with time to reflect on what was learned in the previous session and review the homework. Sessions involved pictures, video clips, and simple activities to increase focus and were designed to be interesting and to encourage participation consistent with the topic of the session. The investigator explained the basic content of each session, and participants shared their feelings and opinions via group discussions, roleplay, and worksheets. Short homework was given for each session, designed to be completed within a half hour to 1 h, for participants to apply what they have learned to their daily life and organize their thoughts in relation to the content of the session.

To increase cohesion, each session was conducted in small groups of three to six participants. The control group received 2 h lectures (school bullying prevention education) provided for adolescents who bully in the participating institutions. To ensure intervention fidelity, the corresponding author delivered the intervention in all the sessions. Furthermore, to control intervention quality, the same author, who is a psychiatric mental health nurse practitioner with 12 years’ experience (including experience in a child and adolescent psychiatric ward as a school bullying project manager), used program manuals and activity worksheets to ensure that the program was delivered consistently.

### 2.6. Outcome Measures

#### 2.6.1. Social and Emotional Competence

Social and emotional competence was measured by the social and emotional function test [31], which was developed for Korean children and adolescents based on a social and emotional assets and resilience scale [32] used to evaluate the social and emotional attitudes of adolescents. This scale consists of four subscales—social competence, emotional regulation, empathy, and self-esteem—and includes a total of 52 items. The items ask about the subject’s typical attitude toward themselves, for example, “I understand the feelings of others well.” Each response is provided using a four-point Likert scale ranging from “not at all” to “always”. Cronbach’s αs were 0.97 in a previous study [31] and 0.97 in the current study. By subscale, social competence was 0.94, emotional regulation was 0.91, empathy was 0.85, and self-esteem was 0.90.

#### 2.6.2. School Bullying Behavior

The participant role questionnaire [33] was composed of 32 items and was developed to assess roles in school bullying situations. These roles include the bully, the victim, the bully/victim, the observer, and the defender. To measure school bullying behavior, 7 items from the participant role questionnaire modified by Soo [34] were used to evaluate the “bully” component. The items evaluate the number of times one exhibited behavior related to school bullying within the last month, for example, “I have hit or kicked someone else”. Responses are provided using a five-point Likert scale ranging from “not at all” to “very much.” Higher scores for the bully’s role indicate a greater degree of perceived school bullying behavior. Cronbach’s αs were 0.75 in a study conducted by Seo [34], and 0.85 in the current study.

#### 2.6.3. Mental Health

The Youth Self-Report (YSR) questionnaire developed by Achenbach [35], as well as a standardized tool [36], was used to measure mental health. In total, 118 items measured self-reported behavior, with responses provided by adolescents aged 11–18 years using a three-point Likert scale ranging from “not at all” to “frequently or very often”. The items consist of evaluating behavior that can estimate various mental health problems and evaluating one’s behavior within the last month. For example, there are items such as “Behavior too young for my age”. Internalizing scales included anxiety/depression, withdrawal/depression, and somatic symptoms, and externalizing scales included violation of rules and aggressive behavior. Cronbach’s αs were 0.93 for the Korean version of the YSR questionnaire in a previous study and 0.95 in the current study.

#### 2.6.4. General Characteristics

For general characteristics of participants, demographic and socioeconomic information collected from the YSR [36] was used and included gender, age, education level, friendship, academic performance level, and socioeconomic status. Self-report was evaluated, and friendship was selected from among bad, moderate, and good friendship, and academic performance and socioeconomic status were evaluated as being at low, middle, and high levels.

### 2.7. Data Analysis

The data were analyzed using IBM SPSS 26.0 (Armonk, NY, USA). T-test, Chi-square, and Fisher’s exact tests to assess the homogeneity of the general characteristics of the experimental and control groups. Normality was verified by the Shapiro–Wilk normality test, and the homogeneity test for the dependent variable was analyzed using the t-test when the normal distribution was satisfied and the Mann–Whitney U-test when the normal distribution was not satisfied. Differences between groups and time interaction effects in social and emotional competence, and mental health that satisfied the normal distribution were analyzed using a linear mixed model (LMM). Differences between groups and time interaction effects in school bullying behavior, which did not satisfy the normal distribution, were analyzed using the generalized estimating equation (GEE). In both LMMs and GEEs, age and gender were treated as covariates to analyze the intervention effect. LMMs consider within-subject correlations in the analysis of repeated measures and are a useful analytical method for controlling for the effects of confounders. As the analysis involves observation values rather than numbers of participants, even if there are missing values, all observations other than the missing values themselves can be included in the analysis [37].

## 3. Results

### 3.1. Homogeneity Verification for General Characteristics and Dependent Variables

Participants’ mean age was 14.41 years (±0.82), 91.5% were boys, and 77.5% maintained good friendships. The proportions of participants with low, middle, and high academic performance levels were 36.6%, 38.0%, and 25.4%. In addition, 87.3% of participants reported middling socioeconomic status. Table 2 presents the results of the test of homogeneity between the experimental and control groups, with respect to general characteristics and the dependent variables. There were no significant differences according to gender, age, friendship, academic performance, or socioeconomic status, and the two groups were comparable in terms of all dependent variables.

### 3.2. Verification of the SECE Program’s Effects

#### 3.2.1. Social and Emotional Competence

The social and emotional competence of the experimental group increased with time, while the control group showed an increase from the pretest to the first posttest and a decrease from the first to the second posttest (Table 3). There was a statistically significant effect of the time × group interaction (F = 6.42, *p* = 0.013). The subscale of social and emotional competence showed that social competence (F = 5.18, *p* = 0.025) and emotional regulation (F = 6.01, *p* = 0.016) also exhibited this trend, which was statistically significant. In addition, the experimental group’s empathy scores increased from the pretest to the first posttest, and were maintained at the second posttest, but the control group showed no significant change over time; therefore, the difference between the two groups was statistically significant (F = 4.79, *p* = 0.031). Self-esteem showed a similar trend to that of empathy, but there was no statistically significant difference between groups (F = 3.45, *p* = 0.066).

#### 3.2.2. School Bullying Behavior

School bullying behavior in the experimental group decreased significantly over time. The control group also showed a decreasing trend over time, but this was smaller relative to that of the experimental group. There was a statistically significant difference in the time × group interaction (Ward χ^2^ = 20.19, *p* < 0.001; Table 3).

#### 3.2.3. Mental Health

The experimental group’s mental health showed a tendency to improve over time. However, there was no statistically significant difference between the experimental and control groups (F = 0.30, *p* = 0.583; Table 3). The results for the internalizing subscale, including anxiety/depression, withdrawal/depression, and somatic symptoms, showed that the experimental group exhibited a decreasing trend in internalizing, but the control group’s scores decreased from the pretest to the first posttest and increased from the first to second posttest. In contrast, the results for the externalizing subscale, including rule violation and aggressive behavior, showed that scores tended to decrease gradually in both the experimental and control groups. However, neither showed statistically significant differences between assessments.

## 4. Discussion

### 4.1. Strength of the SECE Program

In this study, we implemented an SECE program for adolescents who bully to verify its effects on participants’ school bullying behavior and mental health. The predictive factors for school bullying are diverse, and complex factors are generated via interactions [17]. Therefore, adolescents who bully who display the same behaviors have characteristics that could be caused by different factors [18]. The results of the study have implications for the development of integrated interventions based on social and emotional competence, rather than single interventions, to ensure the inclusion of various characteristics of adolescents who bully. Providing a combined program of individual and group interventions could provide an opportunity to complement the negative aspects of group intervention in delinquent adolescents and establish therapeutic relationships.

### 4.2. Effects of the Intervention on Social and Emotional Competence

After the program was provided, the experimental group’s social and emotional competence level was higher relative to that of the control group. This is consistent with the findings of previous studies in which adolescents’ social and emotional competence was elevated through social emotional learning [11,31]. Because the program is designed to help participants to recognize their own emotions and those of others, control their emotions, improve their interpersonal skills, and provide each other with feedback, they were able to control their own emotions and increase their social competence while applying the skills acquired in everyday life. This could be related to the intensive education in empathy and empathic skill training to enhance social awareness in the program. In a study conducted by Şahin [38], who implemented an empathy training program for adolescents who bully, empathy improved after the intervention, and it was similar to that observed at the end of the program at the 2-month follow-up assessment. In the social and emotional competence scale and subscales, the experimental group’s scores showed a tendency to improve or remain constant after the intervention, but the control group showed a temporary improvement after the intervention. Therefore, the program could allow continuous increases in and maintenance of social and emotional competence in adolescents who bully.

### 4.3. Effects of the Intervention on School Bullying Behavior

The experimental group showed a greater decrease in school bullying behavior relative to the control group. These findings were similar to those of a previous study in which social emotional learning prevented and reduced school bullying behavior [11]. This is consistent with the findings of another previous study [26] that implemented emotional literacy programs that improved self-awareness, self-control, empathy, and interpersonal skills by modifying social emotional learning for adolescents who bully. This suggests that enhancing social and emotional competence is effective for not only preventing school bullying in adolescents generally but also reducing school bullying behavior in adolescents who bully. The reduction in bullying behavior by adolescents who bully is of great importance. In addition, it should be noted that school bullying behavior in the experimental group showed a significantly greater reduction relative to that in the control group. Previously, established education was provided for the control group, and their school bullying behavior tended to decrease slightly. However, the experimental group showed a much larger decrease and engaged in almost no school bullying behavior 1 month after the end of the program. In a previous study that examined the role of social emotional learning in preventing school bullying, empathy, emotional management, social problem solving, and social competence, which are part of social and emotional competence, were the main variables that reduced school bullying behavior [39]. The goal of the SECE program is to decrease participants’ school bullying behavior, and it was shown to be a useful nursing intervention for adolescents who bully in this study.

### 4.4. Effects of the Intervention on Mental Health

Mental health in the experimental group showed greater improvements relative to those of the control group, but the difference between groups was not statistically significant. This result was inconsistent with those of previous studies [11,32] in which adolescents’ mental health improved through social emotional learning. The reason for this result is probably related to the timing of the measurement. In previous studies involving mental health promotion, it did not exert significant effects in the short term but was effective in long-term follow-up and at least 6 months after the end of the program [11]. In the present study, although mental health problems in the experimental group showed a tendency to decrease steadily, in the control group, they were alleviated temporarily at the posttest and returned to original levels 1 month later. In addition, a systematic review of children and adolescents who bully showed that school bullying behavior can be maximized if adolescents who bully experience emotional, behavioral, and developmental problems; conduct disorder; oppositional defiant disorder; ADHD; and other mental health problems [17]. Therefore, there is a need for continued attention to improve mental health in adolescents who bully.

### 4.5. Limitations and Future Directions

This study and its results have several limitations. First, due to the study’s quasi-experimental design, participants were not allocated randomly to the experimental and control groups, which likely biased the results and limited generalizability. Randomized controlled trials should be conducted to control for confounding and spurious factors. Second, the program was a short-term intervention lasting for 5 consecutive days from receipt of instructions as compulsory education. However, intensive short-term intervention can prevent dropout and proceed effectively. Since this abbreviated program was found to decrease bullying behavior, studies that apply to various clinical and community environments are needed, as well as a study that verifies the program’s effects. Third, self-reports of school bullying behavior might have led to the results being over-/underestimated. In future studies, it would be beneficial to include objective information such as observer reports and the actual number of reports of school bullying. Finally, we did not conduct a long-term follow-up later than 1 month after completion of the program, meaning we could not observe whether significant improvement in mental health was sustained. We suggest conducting a follow-up assessment at least 6 months after completion of the program so that future studies can investigate this.

## 5. Conclusions

This study developed a social and emotional competence enhancement (SECE) program for adolescents who bully and found a significant positive effect on social competence, emotional regulation, empathy, and school bullying behavior. This program is a short-term intervention consisting of individual interventions based on therapeutic relationships and group interventions including social and emotional learning and cognitive behavioral strategies. A strength of this program is that it can be flexibly implemented by reflecting the reality of the clinical field. School and community-based mental health professionals in a referral setting for adolescents who bully recognize that such adolescent are vulnerable subjects and need to be taken care of. Educators and health professionals for children and adolescents should continue their activities to increase these adolescent’s social and emotional competence.

## Figures and Tables

**Table 1:** SECE program composition.

Session	Theme	Objectives	Contents
1st ^†^	Orientation	a. Increasing overall understanding of the program and SEC b. Forming rapport through individual interview	a. Introducing program objectives and process b. Assessing school bullying experience and mental health status c. Understanding the meaning and importance of SEC d. Setting program rules and establishing contract
2nd	Self-awareness	a. Understanding oneself-needs, values, strengths-through exploring and exposure b. Identify and express emotion	a. Knowing one’s needs and values b. Recognizing strengths in, and mobilizing positive feelings about self, school, family, and support networks c. Recognizing and naming one’s emotions using emotion words d. Understanding the reasons and circumstances for feeling as one does
3rd	Self-management (I) Emotional regulation	a. Understanding the cognitive triangle b. Application of the emotion regulation techniques to daily life	a. Verbalizing and coping with anxiety, depression, and anger b. Understanding the links between thought, emotion, and behavior c. Identify the ‘think trap’ and replace negative thoughts with positive alternatives d. Discuss how to regulate emotions
4th	Self-management (II) Stress management	a. Managing personal and interpersonal stress b. Establishing a coping strategy	a. Understanding the concepts and influence of stress b. Identifying one’s stress, stressor, stressful events, and levels c. Identifying the physical and mental signs of a stress situation d. Finding Healthy and useful coping strategies using a “Stress recipe”
5th	Social awareness (I) Understanding Empathy	a. Understanding diversity and others’ perspectives b. Recognizing others’ emotions c. Increasing empathy	a. Understanding others’ perspectives, points of view, and feelings b. Showing sensitivity to social–emotional cues using a “Ekman’s facial expression” and “Cartoons” c. Thinking from the other side’s perspective d. Understanding victim’s mind in school violence situation
6th	Social awareness (II) Practicing Empathy	a. Increasing listening closely b. Increasing empathic expression	a. Increasing empathy and sensitivity to others’ feelings b. Identifying language expressing empathy c. Practice active empathic listening d. Practice empathic communication skills
7th	Relation skills	a. Enhancing conflict solving skills b. Enhancing communication skills	a. Understanding social conflict and the cause b. Explore conflict resolution c. Identify frequently used languages and exchange negative expressions d. Practice “I-message” e. Developing assertiveness
8th	Responsible decision making (I) Decision making	a. Understanding the importance of decision b. Making reasonable decisions	a. Sharing and discussing our decision recently b. Considering the impact of decision using the “Butterfly effect” c. Reconsidering the regretting decision according to the decision-making process d. Strategy for better decision making
9th	Responsible decision making (II) Goal-setting	a. Setting realistic short- and long-term goals b. Improving responsibility for life by exposing goals	a. Understanding SMART goals: Specific, Measurable, Attainable, Relevant, and Timely b. Setting and sharing goals using a “Roadmap for life” c. Discuss strategies for achieving goals
10th ^†^	Reflection & wrap-up	a. Reflect their own changes through individual interview b. Find social support	a. Review SEC enhancement strategies b. Identify one’s change during the program c. Share specific plans to apply the learned interventions in daily life after the end of program d. Find social support to help keep changes e. Evaluating changes

SEC—Social and emotional competence. ^†^—Individual sessions.

**Table 2:** Homogeneity verification for participants’ general characteristics and dependent variables (*n* = 71).

Characteristic	Category	Total (*n* = 71) *n* (%) or Mean ± SD	Exp. (*n* = 35) *n* (%) or Mean ± SD	Cont. (*n* = 36) *n* (%) or Mean ± SD	t or χ^2^	*p*
Age		14.41 ± 0.82	14.46 ± 0.85	14.36 ± 0.80	0.49	0.626
Gender	Boy	65 (91.5)	33 (94.3)	32 (88.9)	0.67 ^†^	0.674
	Girl	6 (8.5)	2 (5.7)	4 (11.1)		
Friendship	Bad	0 (0)	0 (0.0)	0 (0.0)	0.00	0.949
	Moderate	16 (22.5)	8 (22.9)	8 (22.2)		
	Good	55 (77.5)	27 (77.1)	28 (77.8)		
Academic performance	Low	26 (36.6)	14 (40.0)	12 (33.3)	2.47	0.290
	Middle	27 (38.0)	15 (42.9)	12 (33.3)		
	High	18 (25.4)	6 (17.1)	12 (33.3)		
SES	Low	5 (7.0)	3 (8.6)	2 (5.6)	1.44 ^†^	0.486
	Middle	62 (87.3)	29 (82.9)	33 (91.7)		
	High	4 (5.6)	3 (8.6)	1 (2.8)		
Dependent Variables		Total (*n* = 71) Mean ± SD	Exp. (*n* = 35) Mean ± SD	Cont. (*n* = 36) Mean ± SD	Z	*p*
Social and emotional competence	Total	139.38 ± 27.82	133.43 ± 24.71	145.17 ± 9.74	−1.84	0.066
	Social Competence	58.23 ± 12.35	55.69 ± 11.25	60.69 ± 13.01	−1.73	0.084
	Emotional Regulation	29.97 ± 7.14	28.66 ± 6.24	31.25 ± 7.79	−1.45	0.147
	Empathy	18.52 ± 4.09	17.71 ± 4.03	19.31 ± 4.04	−1.70	0.089
	Self-esteem	32.66 ± 6.80	32.66 ± 6.80	33.92 ± 7.50	−1.64	0.102
School bullying behavior		8.68 ± 5.82	9.91 ± 5.89	7.47 ± 5.57	−1.87	0.062
Mental health	Total	45.08 ± 9.43	46.94 ± 9.16	43.28 ± 9.47	−1.81	0.070
	Internalizing	42.63 ± 9.38	43.97 ± 9.55	41.33 ± 9.17	−1.20	0.263
	Externalizing	50.13 ± 11.46	51.51 ± 9.57	48.78 ± 13.03	−0.97	0.319

^†^—Fisher’s exact test; Exp.—Experimental group; Cont.—Control group; SD—standard deviation; SES—Socioeconomic status.

**Table 3:** Intervention effects on dependent variables (*n* = 71).

Outcomes	Category	Time	Exp. (*n* = 35)	Cont. (*n* = 36)	Time		Group		Group × Time	
			M ± SD	M ± SD	F or Wald χ^2^	*p*	F or Wald χ	*p*	F or Wald χ	*p*
Social and emotional competence	Total	Pre	133.43 ± 24.71	145.17 ± 29.74	11.84	0.001	6.07	0.016	6.42	0.013
		Post I	147.40 ± 30.98	152.03 ± 33.20						
		Post II	150.09 ± 35.22	146.12 ± 33.81						
	Social Competence	Pre	55.69 ± 11.25	60.69 ± 13.01	5.88	0.017	5.24	0.024	5.18	0.025
		Post I	61.00 ± 13.25	63.56 ± 14.58						
		Post II	61.94 ± 14.55	60.42 ± 14.17						
	Emotional Regulation	Pre	28.66 ± 6.24	31.25 ± 7.79	19.25	<0.001	5.06	0.027	6.01	0.016
		Post I	31.57 ± 8.09	33.11 ± 8.23						
		Post II	33.71 ± 8.96	32.15 ± 8.20						
	Empathy	Pre	17.71 ± 4.03	19.31 ± 4.04	5.95	0.017	3.99	0.049	4.79	0.031
		Post I	19.86 ± 4.59	19.81 ± 4.71						
		Post II	19.74 ± 5.47	19.39 ± 5.10						
	Self-esteem	Pre	31.37 ± 5.83	33.92 ± 7.50	6.44	0.013	4.11	0.046	3.45	0.066
		Post I	34.34 ± 7.58	35.56 ± 7.52						
		Post II	34.71 ± 8.04	34.15 ± 8.46						
School bullying behavior		Pre	9.91 ± 5.89	7.47 ± 5.57	93.29	<0.001	0.06	0.804	20.19	<0.001
		Post I	7.43 ± 5.56	6.28 ± 5.40						
		Post II	2.74 ± 3.70	5.48 ± 5.91						
Mental health	Total	Pre	46.94 ± 9.16	43.28 ± 9.47	11.45	0.001	3.12	0.081	0.30	0.583
		Post I	44.29 ± 12.00	39.28 ± 10.79						
		Post II	41.25 ± 10.64	41.86 ± 14.08						
	Internalizing	Pre	43.97 ± 9.55	41.33 ± 9.17	5.08	0.027	3.16	0.079	1.83	0.179
		Post I	41.17 ± 9.35	39.06 ± 8.56						
		Post II	39.33 ± 11.37	40.93 ± 15.55						
	Externalizing	Pre	51.51 ± 9.57	48.78 ± 13.03	7.83	0.007	1.11	0.295	0.13	0.909
		Post I	49.54 ± 13.76	44.86 ± 14.44						
		Post II	46.67 ± 11.37	42.90 ± 13.44						

Exp.—Experimental group; Cont.—Control group; SD—standard deviation; Post I—immediately after intervention; Post II—1-month follow-up. Covariates: age, gender.

## Data Availability

The data presented in this study are available on request from the corresponding author. The data are not publicly available due to ethical restriction.
